# Systolic Pulmonary Artery Pressure and Cardiovascular Biomarkers—New Non-Invasive Ways to Detect Pulmonary Hypertension in Patients with Severe Aortic Valve Stenosis Undergoing TAVR?

**DOI:** 10.31083/j.rcm2307224

**Published:** 2022-06-24

**Authors:** Elke Boxhammer, Clara Köller, Vera Paar, Dzeneta Fejzic, Richard Rezar, Christian Reiter, Jürgen Kammler, Jörg Kellermair, Matthias Hammerer, Hermann Blessberger, Clemens Steinwender, Uta C. Hoppe, Michael Lichtenauer

**Affiliations:** ^1^Division of Cardiology, Department of Internal Medicine II, Paracelsus Medical University of Salzburg, 5020 Salzburg, Austria; ^2^Department of Cardiology, Kepler University Hospital, Medical Faculty of the Johannes Kepler University Linz, 4020 Linz, Austria

**Keywords:** aortic valve stenosis, biomarker, pulmonary hypertension, systolic pulmonary artery pressure

## Abstract

**Background:**

Patients with severe aortic valve stenosis (AS) frequently present with pulmonary hypertension (PH). The gold standard for detection of pulmonary hypertension is right heart catheterization, which is not routinely performed as a preoperative standard in cardiology centers today, neither before surgical valve replacement nor before transcatheter aortic valve replacement (TAVR) procedure. Echocardiographic determination of systolic pulmonary artery pressure (sPAP) provides an opportunity to assess the presence or absence of PH. The aim of the present study was to investigate the extent to which plasma levels of common cardiovascular biomarkers behave in patients with severe AS and an sPAP <40 mmHg in comparison to patients with an sPAP ≥40 mmHg.

**Methods:**

179 patients with echocardiographic evidence of severe AS before TAVR procedure were divided into 2 groups based on sPAP. An sPAP of 40 mmHg was considered the cut-off value, with absence of PH defined by an sPAP <40 mmHg (n = 82) and presence of PH defined by an sPAP ≥40 mmHg (n = 97). Directly before TAVR, a blood sample was drawn from each patient, and plasma concentrations of the cardiovascular biomarkers Soluble Suppression of Tumorigenicity-2 (sST2), Growth/Differentiation of Factor-15 (GDF-15), Heart-Type Fatty-Acid Binding Protein (H-FABP), Insulin Like Growth Factor Binding Protein 2 (IGF-BP2), Soluble Urokinase-Type Plasminogen Activator Receptor (suPAR), Brain Natriuretic Peptide (BNP) and Cardiac Troponin I (cTnI) were determined.

**Results:**

Patients with an sPAP ≥40 mmHg had significantly higher sST2 (*p* = 0.010), GDF-15 (*p* = 0.005), IGF-BP2 (*p* = 0.029), suPAR (*p* = 0.018), BNP (*p *< 0.001) and cTnI (*p* = 0.039) plasma levels. Only for H-FABP (*p* = 0.069), no significant differences were discernible between the two groups. In addition, cut-off values were calculated to predict an sPAP ≥40 mmHg. Significant results were shown with 16045.84 pg/mL for sST2 (*p* = 0.010), with 1117.54 pg/mL for GDF-15 (*p* = 0.005), with 107028.43 pg/mL for IGF-BP2 (*p* = 0.029), with 3782.84 pg/mL for suPAR (*p* = 0.018), with 2248.00 pg/mL for BNP (*p *< 0.001) and with 20.50 pg/mL for cTnI (*p* = 0.002).

**Conclusions:**

sPAP as an echocardiographic parameter in combination with supplementary use of cardiovascular biomarkers presented here have the potential to provide more detailed information about the presence or absence of PH in a non-invasive way.

## 1. Introduction

In 48–75% of patients, severe aortic valve stenosis (AS) is associated with pulmonary hypertension (PH), limiting the long-term survival of these patients [1, 2, 3, 4]. The pathophysiological cause is progressive concentric hypertrophy of the left ventricle, which leads to a decrease of compliance and relaxation, thus limiting diastolic function. This leads to an increase of enddiastolic left ventricular filling pressure and subsequently an increase of pulmonary venous pressure. In the course of the disease this may be further aggravated by secondary mitral regurgitation. This causes a so-called vascular remodeling and thus a consecutive pressure increase in the pulmonary arteries, the post-capillary PH.

According to current European Society for Cardiology (ESC) guidelines [5], the gold standard for the detection of PH is and remains invasive right heart catheterization with determination of mean pulmonary artery pressure (mPAP) and pulmonary artery wedge pressure (PAWP). By definition, PH is not present if mPAP <25 mmHg, whereas PH can be assumed if mPAP ≥25 mmHg. This procedure is no longer routinely used as a preoperative diagnostic tool before surgical valve replacement or transcatheter aortic valve replacement (TAVR) in patients with severe AS.

Echocardiography plays a crucial role in obtaining non-invasive information about the possible presence of PH. As a measure of the existence of PH, systolic pressure gradient derived over a tricuspid valve regurgitation, plus estimated right atrial pressure (RAP), provide information. A systolic pulmonary artery pressure (sPAP) ≥40 mmHg is associated with a significantly increased risk of pulmonary hypertension and is therefore used as a relevant cut-off value [6]. However, this parameter is subject to potential error (correct estimation of RAP using inferior vena cava diameter, correct plumbing of maximal regurgitation velocity across the tricuspid valve, echocardiography qualities).

To strengthen the significance of sPAP as an important non-invasive parameter, in the present study, patients with severe AS planned for TAVR procedure were examined echocadiographically for the potential presence of PH (sPAP ≥40 mmHg) and the expression of various cardiovascular biomarkers was assessed in relation to sPAP.

### 1.1 Soluble Suppression of Tumorigenicity-2 (sST2) 

Suppression of tumorigenicity (ST2) belongs to the Toll-like/IL-1 receptor family and exists in two different forms, one as a transmembrane form (ST2L) and the other as a soluble form (sST2) [7]. ST2L interacts with IL-33 as a ligand-receptor complex and acts in a complex signaling cascade against cardiac remodeling and fibrotic remodeling processes. However, high mechanical stress responses in the heart and lungs result in increased secretion of sST2 from alveolar epithelial cells and cardiac myocytes. These bind with higher affinity to Interleukin (IL)-33 and thus prevent cardioprotective signaling. High plasma sST2 levels are therefore associated with an increased risk of adverse outcomes in patients with severe AS, heart failure and PH [8, 9].

### 1.2 Growth/Differentiation of Factor-15 (GDF-15)

GDF-15 is a member of the transforming growth factor-beta (TGF-β) superfamily and is secreted by numerous cells such as macrophages, cardiomyocytes, pulmonary endothelial cells and vascular smooth muscle cells [10, 11]. The growth factor plays a crucial role in inflammatory processes, tissue injury and apoptosis and has been found to be eleveated in numerous pathological conditions such as cardiovascular and pulmonary disease [12, 13].

### 1.3 Heart-Type Fatty-Acid Binding Protein (H-FABP)

H-FABP is a cytoplasmic protein secreted by cardiomyocytes in the context of acute ischemic heart disease. At the molecular level, H-FABP is involved in lipid metabolism, transporting fatty acids from the cell membrane to mitochondria for eventual oxidation [14]. This biomarker has already found its way into clinical practice, as it is already available as a rapid test to diagnose myocardial infarction at an earlier stage [15].

### 1.4 Insulin Like Growth Factor Binding Protein 2 (IGF-BP2)

IGF-BP2 is an important member of the insulin-like growth factor family regulating the activity of the insulin-like growth factor (IGF) in most tissues and organs including liver, heart, CNS and reproductive organs [16]. IGF-BP2 exerts an inhibitory effect on the growth hormone IGF-1, which has a cardioprotective function by downregulating the renin-angiotensin-aldosterone system [17]. Elevated IGF-BP2 levels, through IGF-1 inhibition, thus lead to a consecutive unopposed renin-angiotensin-aldosterone effect, resulting in cardiac remodeling and left ventricular dysfunction [18].

### 1.5 Soluble Urokinase-Type Plasminogen Activator Receptor (suPAR)

suPAR is the soluble form of the cell membrane protein urokinase-type Plasminogen Activator Receptor (uPAR), is released into the blood during inflammation of any kind and therefore provides information about inflammatory activity in the human body. Numerous studies have described increased plasma concentrations in patients with coronary heart disease, myocardial infarction and chronic heart failure [19].

### 1.6 Brain Natriuretic Peptide (BNP)

BNP is a cardiac hormone, which is released by cardiomyocytes in the course of stretching processes of the left ventricle. Especially in patients with pressure and volume overload, the plasma concentration of BNP is significantly increased. Evidence based, it is already handled as a relevant heart failure biomarker in clinical practice. Patients with moderate to severe AS showed significantly increased mortality at higher BNP plasma concentrations compared to patients with baseline BNP at follow-up [20].

### 1.7 Cardiac Troponin I (cTnI)

Troponin is a relevant protein complex consisting primarily of three subunits. Two of them, troponin T and troponin I are specifically formed in the myocardium and are relevantly involved in the interaction of actin and myosin filaments. Any form of damage to cardiac myocytes will result in increased release of troponin. This is exploited clinically for early detection of myocardial infarction [21].

## 2. Material & Methods

### 2.1 Study Population

Between 2016 and 2018, 179 patients with severe, primary degenerative AS planning for TAVR procedure were enrolled in current study. Corresponding data analyses were performed at Paracelsus Medical University Hospital Salzburg and Kepler University Hospital Linz in accordance to to principles of the Declaration of Helsinki and Good Clinical Practice.

### 2.2 Transthoracic Echocardiography

Transthoracic echocardiography was performed using common ultrasound devices (iE33 and Epiq 5; Philips Healthcare, Hamburg, Germany). Severe AS was classified according to current valid guidelines of European Society for Cardiology measuring. An AV Vmax (maximal velocity over aortic valve) of 4.0 m/s, an AV dpmean (mean pressure gradient over aortic valve) ≥40 mmHg and an aortic valve area ≤1.0 ${\mathrm{cm}}^{2}$ formed the definition of severe AS. Patients with low-flow, low-gradient AS and a stroke volume <35 mL/$m^{2}$ were excluded from the study, so the patient population presented here includes solitary individuals with high pressure gradients without a low-flow situation. Simpson’s method was applied to receive left ventricular ejection fraction (LVEF). To graduate mitral, aortic, and tricuspid valve regurgitation in minimal, mild (I), moderate (II) and severe (III) spectral and color-Doppler images were used. The maximum tricuspid regurgitant jet velocity (TRV) is obtained by continuous wave Doppler over the tricuspid valve and is used to calculate the pulmonary artery pressure (PAP) using the formula 4 × ${\mathrm{TRV}}^{2}$. To finally obtain the sPAP, which is decisive for PH, the right atrial pressure (RAP) had to be estimated. This corresponds to the central venous pressure and is determined by the diameter of the inferior vena cava (IVC). The following RAP determination was performed in the respective cohorts from Salzburg and Linz: With an IVC diameter ≥21 mm and a respiratory caliber fluctuation <50%, a RAP of 15 mmHg was assumed. For an IVC diameter <21 mm as well as a respiratory caliber fluctuation ≥50%, a RAP of 3 mmHg was calculated. Other scenarios not corresponding to the above constellations were ascribed an intermediate value of 8 mmHg. Finally, the simplified Bernoulli equation (4 × ${\mathrm{TRV}}^{2}$) + RAP leads to an sPAP result. An sPAP ≥40 mmHg was used as the cut-off value to determine PH in accordance with the current literature [22, 23, 24]. In particular, Schewel *et al*. [25] compared echocardiographically obtained sPAP with invasively RHC obtained sPAP data. The correlation coefficient of r = 0.820 was in a very satisfactory range. This study also demonstrated that a cut-off value ≥40 mmHg had better overall statistical goodness criteria than a cut-off value ≥45 mmHg or ≥50 mmHg.

### 2.3 Biomarker Analysis

Blood samples were obtained on the day of hospitalization and thus one day before the actual TAVR procedure under fasting conditions using a vacuum-containing system. The collection tubes were centrifuged, the plasma obtained was separated from the blood components and then frozen at –80 °C to analyze the total of 179 samples at similar time points under same conditions.

Plasma levels of sST2, GDF-15, H-FABP, IGF-BP2 and suPAR were measured by using enzyme-linked immunosorbent assay (ELISA) kits (sST2: Duoset DY523, GDF-15: DY957, H-FABP: DY1678, IGF-BP2: DY674, suPAR: DY807, R&D Systems, Minneapolis, MN, USA). Instructions of the manufactures were performed for adequate preparation of reagents. In summary, serum samples and standard protein were loaded onto the wells of ELISA plates (Nunc MaxiSorp flat-bottom 96 well plates, VWR International GmbH, Vienna, Austria) and incubated for two hours. The plates were treated with Tween 20/PBS solution (Sigma Aldrich, St. Louis, MO, USA) and subsequently a biotin-labeled antibody was added. The subsequent incubation time was another two hours. A washing process again was performed and streptavidin-horesradish-peroxidase solution was added to the wells. A color reaction was generated after adding tetramethylbenzidine (TMB; Sigma Aldrich, St. Louis, MO, USA). Optical density was determined at 450 nm on an ELISA plate-reader (iMark Microplate Absorbance Reader, Bio-Rad Laboratories, Vienna, Austria).

### 2.4 Statistical Analysis

Statistical analysis was performed using SPSS (Version 25.0, SPSS Inc., Armonk, NY, USA).

First of all, the Kolmogorov-Smirnov test was applied to test variables for normal distribution. Normally distributed metric data was expressed as mean ± standard deviation (SD) and analyzed using an unpaired student’s *t*-test. Not-normally distributed metric data was expressed as median and interquartile range (IQR) and the Mann-Whitney-U-test was applied for statistical analysis. Frequencies/percentages were used for categorial clinical data and compared using the chi-square test.

To determine an optimal cut-off value of examined cardiovascular biomarkers according to a prediction of an sPAP ≥40 mmHg, Area Under the Receiver Operator Characteristics (AUROC)-curves with Area Under the Curve (AUC) and separate analysis of Youden Index (YI) was performed.

Correlation analysis was performed using Pearson’s rank-correlation coefficient to draw conclusions about a relationship between echocardiographic sPAP and cardiovascular biomarkers.

Kaplan-Meier curves were carried out to detect overall 1-year survival of patients in dependence of sPAP, whereby the currently accepted classification into three severity levels (I: sPAP <40 mmHg, II: sPAP 40–59 mmHg; III: sPAP ≥60 mmHg) was used.

At last, a univariate Cox proportional hazard regression model was used to calculate hazard ratio (HR) and 95% confidence interval (CI) for several influencing factors associated with 1-year-mortality in patients undergoing TAVR procedure. For better comparability, a z-transformation was absolved for metric data. Afterwards, multivariate Cox regression was performed to assess independent predictors of mortality. Therefore, again covariates associated with mortality in the univariate analysis (*p <* 0.100) were entered and a backward variable elimination was done.

A *p*-value < 0.050 was considered statistically significant.

## 3. Results

### 3.1 Study Cohort

A total of 179 patients with severe AS from the University Hospitals of Salzburg and Linz were included in the study. Echocardiographically, 82 patients (45.8%) showed an sPAP <40 mmHg equivalent to the absence of PH and 97 patients (54.2%) showed an sPAP ≥40 mmHg consistent with the echocardiographic criterion of PH.

### 3.2 Baseline Characteristics of the Study

Table 1 shows the collected baseline characteristics of the overall cohort as well as those of the classification into patients with sPAP <40 mmHg and sPAP ≥40 mmHg. The sPAP groups were compared with each other for significance and the corresponding *p*-values were documented.

**Table 1.** **Patient characteristics of study cohort**.

	Overall cohort		sPAP <40 mmHg		sPAP ≥40 mmHg		
	n = 179		n = 82		n = 97		
Clinical data							*p*-value
Age (years) - mean ± SD	82.7	4.8	81.6	4.8	83.7	4.6	0.277
Gender (male) - %	50.8		51.2		50.5		0.925
Weight (kg) - mean ± SD	71.5	11.1	73.1	14.8	70.6	6.0	**0.003**
Height (cm) - mean ± SD	166.2	7.3	165.1	9.2	166.8	3.0	0.556
BMI (kg/$m^{2}$) - mean ± SD	25.9	3.7	27.1	4.5	25.1	3.0	0.518
NYHA - median ± IQR	3.0	1.0	3.0	1.0	3.0	0.8	0.122
STSScore - mean ± SD	3.0	1.5	2.6	1.3	3.3	1.5	**0.025**
Concomitant disease							*p*-value
Diabetes mellitus - %	23.5		20.7		25.8		0.428
Arterial Hypertension - %	78.8		76.8		80.4		0.559
CVD - %	72.1		73.2		71.1		0.847
CVD - 1 vessel - %	23.5		24.4		22.7		0.891
CVD - 2 vessels - %	8.4		4.9		11.3		0.103
CVD - 3 vessels - %	11.7		9.8		13.4		0.398
Myocardial infarction - %	3.4		2.4		4.1		0.542
Atrial fibrillation - %	38.0		28.0		46.4		**0.012**
Pacemaker - %	6.7		4.9		8.2		0.369
Malignancy - %	21.2		24.4		18.6		0.342
Stroke - %	6.7		6.1		7.2		0.768
pAVK - %	5.6		3.7		7.2		0.302
COPD - %	9.5		8.5		10.3		0.687
Echocardiography							*p*-value
LVEF (%) - mean ± SD	55.0	10.9	56.7	8.6	53.6	12.4	0.054
LVEDD (mm) - mean ± SD	5.1	4.1	4.6	0.7	5.4	5.2	0.402
IVSd (mm) - mean ± SD	15.0	3.0	14.9	3.0	15.0	2.9	0.761
AV Vmax (m/s) - mean ± SD	4.6	3.0	4.4	0.6	4.9	4.2	0.301
AV dPmean (mmHg) - mean ± SD	49.5	12.6	48.4	11.8	50.5	13.3	0.300
AV dPmax (mmHg) - mean ± SD	79.5	19.4	78.2	18.0	80.7	20.7	0.407
TAPSE (mm) - mean ± SD	21.7	3.8	23.1	3.2	20.8	3.9	**0.008**
AVI ≥II° - %	16.2		17.1		15.5		0.868
MVI ≥II° - %	25.1		15.9		33.0		**0.009**
TVI ≥II° - %	18.4		6.1		28.9		< **0.001**
Laboratory data							*p*-value
Creatinine (mg/dL) - median ± IQR	1.0	0.4	0.9	0.3	1.1	0.5	**0.015**
BNP (pg/mL) - median ± IQR	2020.0	3879.2	1195	1024.2	3369	4978	< **0.001**
cTnI (pg/mL) - median ± IQR	23.0	19.8	16.0	13.5	27.0	18.5	**0.039**
Hkt (%) - median ± IQR	38.2	8.9	41.2	6.7	37.4	8.9	**0.019**
Hb (g/dL) - median ± IQR	12.7	2.5	13.1	2.3	12.3	3.2	**0.014**
CK (U/L) - median ± IQR	59.0	73.0	74.0	117.0	59.0	68.8	0.220
sST2 (pg/mL) - median ± IQR	13847.7	8084.5	11563.7	6708.6	16467.1	10606.6	**0.010**
GDF-15 (pg/mL) - median ± IQR	638.8	1000.4	357.2	683.2	785.9	1034.8	**0.005**
H-FABP (ng/mL) - median ± IQR	0.5	1.9	0.4	1.3	0.5	2.4	0.069
IGF-BP2 (pg/mL) - median ± IQR	145518.7	150848.9	94235.2	137450.8	203352.4	169893.4	**0.029**
suPAR (pg/mL) - median ± IQR	3458.1	1682.7	3000.5	1127.4	3951.6	1468.3	**0.018**

sPAP, systolic pulmonary artery pressure; BMI, body mass index; CVD, cardiovascular disease; LVEF, left ventricular ejection fraction; LVEDD, left ventricular end-diastolic diameter; IVSd, interventricular septal thickness at diastole; AV Vmax, maximal velocity over aortic valve; AV dpmean, mean pressure gradient over aortic valve; AV dpmax, maximal pressure gradient over aortic valve; TAPSE, tricuspid annular plane systolic excursion; AVI, aortic valve insufficiency; MVI, mitral valve insufficiency; TVI, tricuspid valve insufficiency; BNP, brain natriuretic peptide; cTnI, cardiac Troponin I; CK, creatine kinase; sST2, soluble suppression of tumorigenicity-2; GDF-15, growth/fifferentiation of factor-15; H-FABP, heart-type fatty-acid binding protein; IGF-BP2, insulin like growth factor binding protein 2; suPAR, soluble urokinase-type plasminogen activator receptor; SD, standard deviation; IQR, interquartile range.

The overall cohort had a mean age of 82.7 ± 4.8, with a male:female ratio of 50.8% vs. 48.2%. Regarding concomitant disease, arterial hypertension was documented in 78.8%, general cardiovascular disease in 72.1% and diabetes mellitus in 23.5% of patients. Echocardiographic left ventricular ejection fraction (LVEF) averaged 55.0 ± 10.9% and sPAP 44.6 ± 15.2 mmHg.

Patients with an sPAP ≥40 mmHg, in contrast to those with an sPAP <40 mmHg, showed significantly higher STSScores (3.3 ± 1.5 vs. 2.6 ± 1.3; *p* = 0.025), significantly higher percentages of atrial fibrillation (46.4% vs. 28.0%; *p* = 0. 012), significantly lower tricuspid annular plane systolic excursion (TAPSE) (20.8 ± 3.9 mm vs. 23.1 ± 3.2 mm; *p* = 0.008) and ultimately significantly higher percent distributions with respect to mitral valve (33.0% vs. 15.9%; *p* = 0.009) and tricuspid valve insufficiency (28.9% vs. 6.1%; *p *< 0.001).

### 3.3 Biomarker Concentrations

**Fig. 1.**
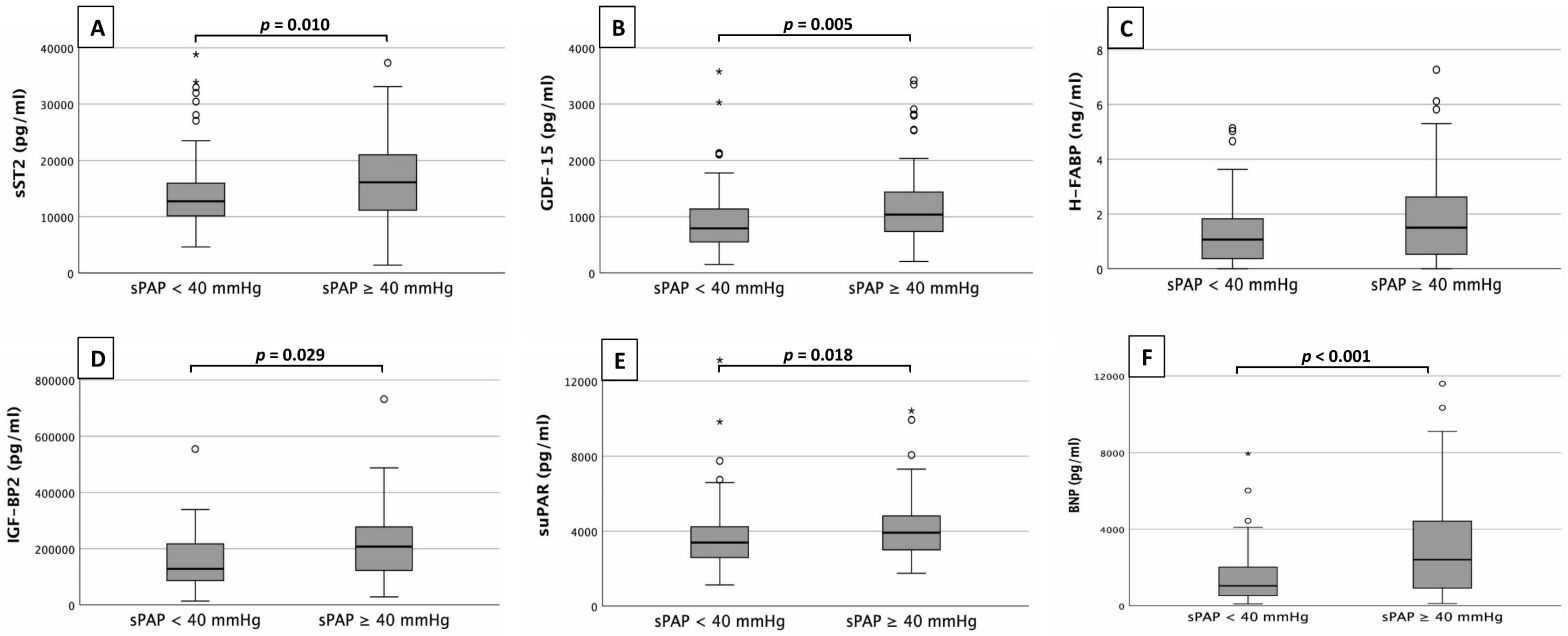
**Biomarker analysis**. Serum concentrations of sST2 (A), GDF-15 (B), H-FABP (C), IGF-BP2 (D), suPAR (E) and BNP (F) in patients with an sPAP <40 mmHg and with an sPAP ≥40 mmHg.

Fig. 1 provides an overview of the corresponding plasma concentrations of the determined cardiovascular biomarkers depending on the sPAP obtained (≥40 mmHg vs. <40 mmHg).

sST2 (Fig. 1A) showed significantly higher plasma concentrations in patients with an sPAP ≥40 mmHg than with an sPAP <40 mmHg (16467.1 ± 10606.6 pg/mL vs. 11563.7 ± 6708.6 pg/mL; *p* = 0.010). Similar constellations were found for GDF-15 (Fig. 1B) with plasma concentrations of 785.9 ± 1034.8 pg/mL vs. 357.2 ± 683.2 pg/mL (*p* = 0.005), IGF-BP2 (Fig. 1D) with plasma concentrations of 203352.4 ± 169893.4 pg/mL vs. 94235.2 ± 137450.8 pg/mL (*p* = 0.029) and suPAR (Fig. 1E) with plasma concentrations of 3951.6 ± 1468.3 pg/mL vs. 3000.5 ± 1127.4 pg/mL (*p* = 0.018). BNP (Fig. 1F) with plasma concentrations of 3369.0 ± 4978.0 pg/mL vs. 1195.0 ± 1024.2 pg/mL (*p *< 0.001) showed the best result among all biomarkers studied. cTnI presented with plasma concentrations of 27.0 ± 18.5 pg/mL vs. 16.0 ± 13.5 pg/mL also statistically significant (*p* = 0.039).

Only H-FABP (Fig. 1C) did not show significant differences between the sPAP groups (0.5 ± 2.4 ng/mL vs. 0.4 ± 1.3 ng/mL; *p* = 0.069).

### 3.4 AUROC Results

**Fig. 2.**
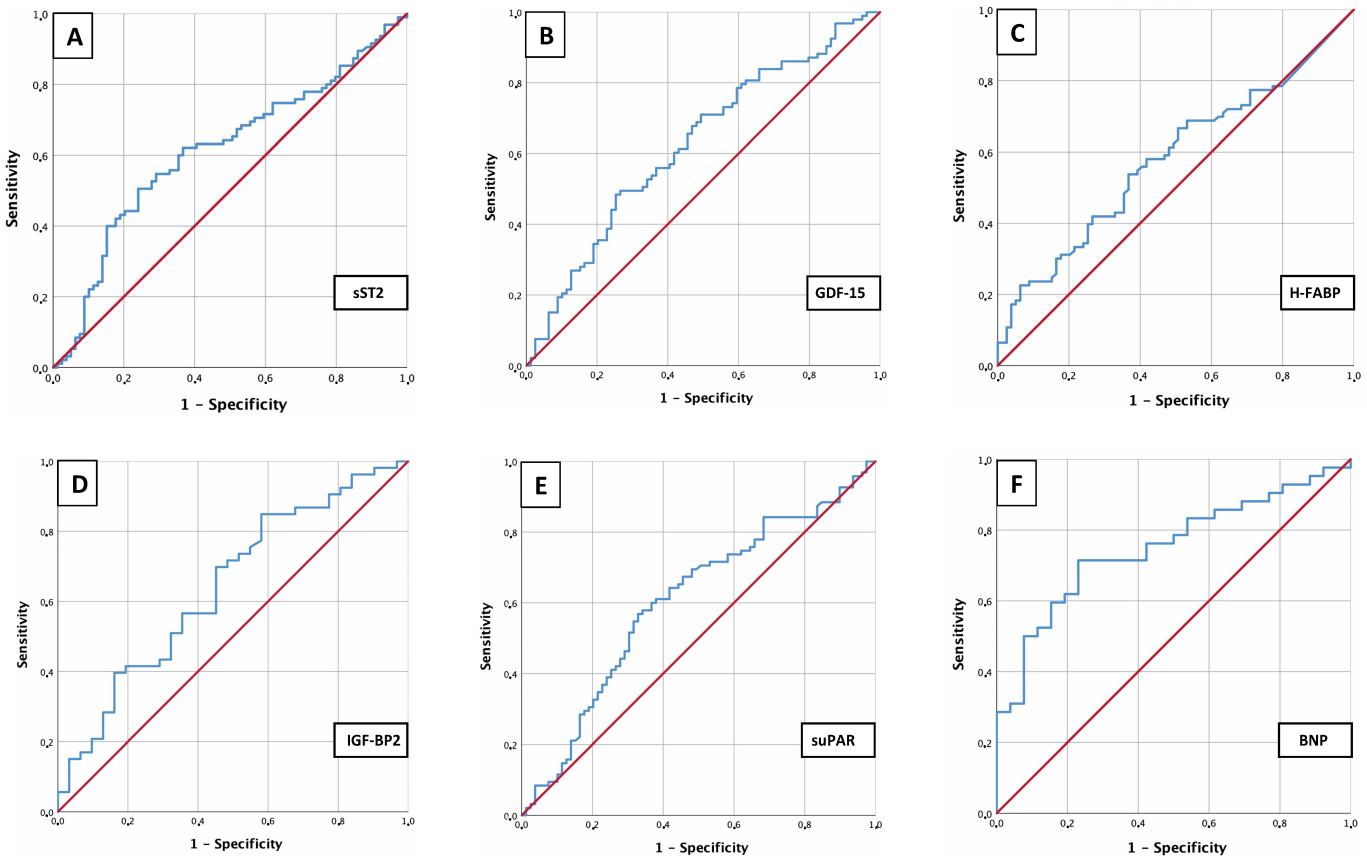
**AUROC analysis of biomarkers**. AUROC analyses of sST2 (A), GDF-15 (B), H-FABP (C), IGF-BP2 (D), suPAR (E) and BNP (F) for prediction of sPAP ≥40 mmHg with concerning cut-off values, Youden Index, sensitivity and specificity.

To analyze sST2, GDF-15, H-FABP, IGF-BP2 and suPAR as potential biomarkers for prediction of an sPAP ≥40 mmHg in patients with severe AS before TAVR, AUROC-curves regarding plasma level concentration of examined biomarkers were figured out. Therefore AUC, cut-off values with YI as well as sensitivity and specificity were extracted in addition to ROC curves (Fig. 2).

This analysis identified an sST2 plasma level (Fig. 2A) of 16045.84 pg/mL as an optimal cut-off value concerning an sPAP ≥40 mmHg (AUC 0.613; 95% CI 0.529–0.698; *p* = 0.010; YI 0.26; sensitivity 0.51; specificity 0.76) (Fig. 2A). GDF-15 (Fig. 2B) provided a cut-off value of 1117.54 pg/mL (AUC 0.624; 95% CI 0.541–0.708; *p* = 0.005; YI 0.23; sensitivity 0.48; specificity 0.75), IGF-BP2 (Fig. 2D) a cut-off value of 107028.43 pg/mL (AUC 0.643; 95% CI 0.520–0.766; *p* = 0.029; YI 0.27; sensitivity 0.85; specificity 0.42), suPAR (Fig. 2E) a cut-off value of 3782.84 pg/mL (AUC 0.605; 95% CI 0.520–0.690; *p* = 0.018; YI 0.24; sensitivity 0.57; specificity 0.67), BNP (Fig. 2F) a cut-off value of 2248.00 pg/mL (AUC 0.692; 95% CI 0.611–0.773; *p *< 0.001; YI 0.36; sensitivity 0.55; specificity 0.82) and cTnI (not graphically shown) a cut-off value of 20.50 pg/mL (AUC 0.704; 95% CI 0.584–0.825; *p* = 0.002; YI 0.36; sensitivity 0.72; specificity 0.65). Merely the determined cut-off value of H-FABP (Fig. 2C) with 1.44 did not provide a significant result with a *p* = 0.070. All in all, the investigated biomarkers showed a rather moderate sensitivity and specificity, with BNP and cTnI performing best in a single biomarker determination.

### 3.5 Pearson’s Correlation

**Fig. 3.**
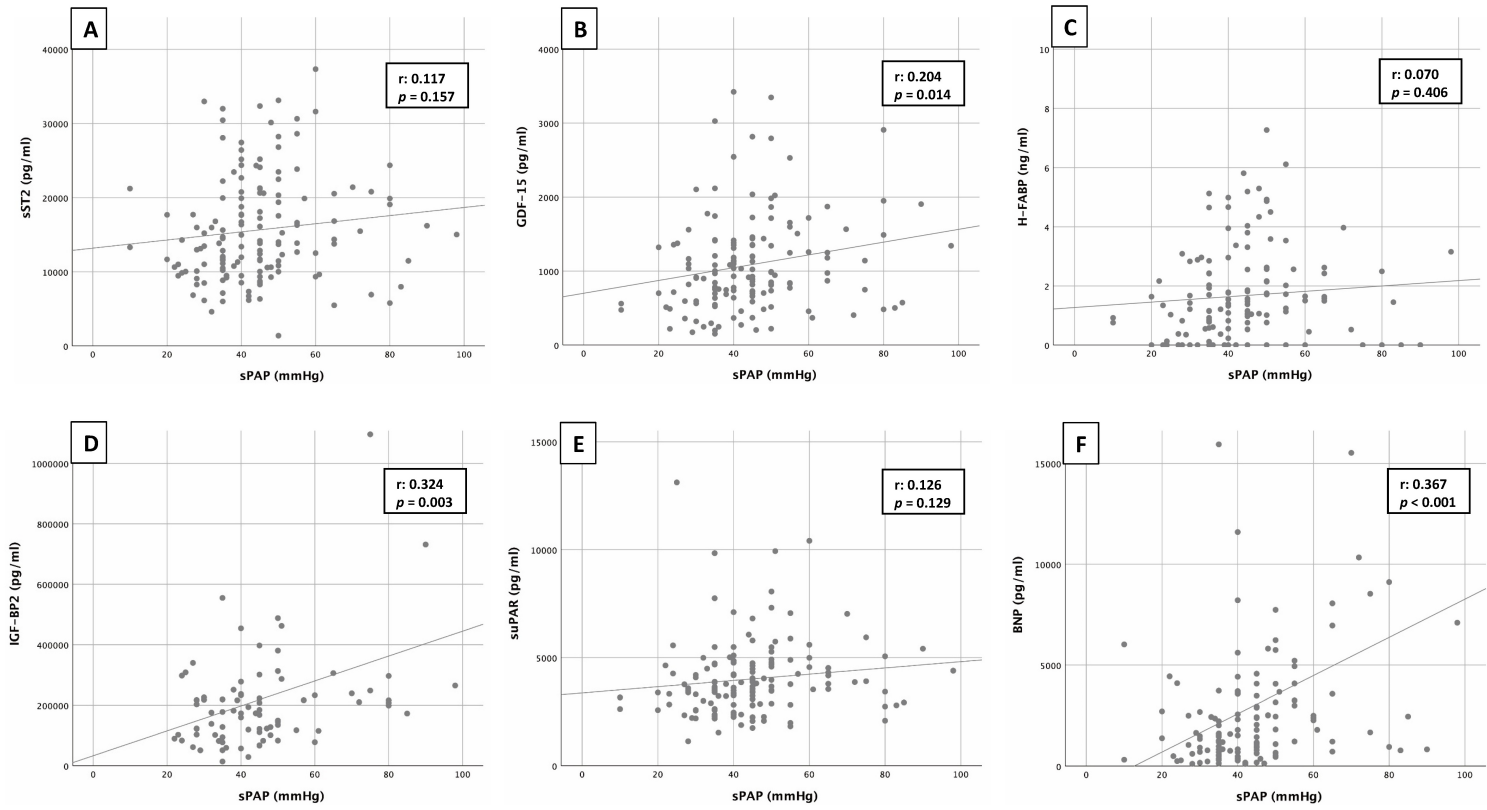
**Correlation of sPAP and biomarkers**. Correlation analyses between sPAP and cardiovascular biomarkers of sST2 (A), GDF-15 (B), H-FABP (C), IGF-BP2 (D), suPAR (E) and BNP (F).

Pearson’s correlation analysis between sPAP and the corresponding biomarkers sST2, GDF-15, H-FABP, IGF-BP2, suPAR and BNP is shown in Fig. 3. Pearson’s correlation coefficient (r) was used to describe potential associations.

Correlation analysis revealed a significant, but moderate linear relationship between sPAP and BNP (r: 0.367; *p *< 0.001) or between sPAP and IGF-BP2 (r: 0.324; *p* = 0.003) and a significant, but weak linear relationship between sPAP and GDF-15 (r: 0.204; *p* = 0.014). The other biomarkers such as sST2 (r: 0.117; *p* = 0.157), H-FABP (r: 0.070; *p* = 0.406), suPAR (r: 0.126; *p* = 0.129) and cTnI (r: 0.174; *p* = 0.115; not graphically demonstrated) showed no significant correlations.

### 3.6 Kaplan-Meier Curves

**Fig. 4.**
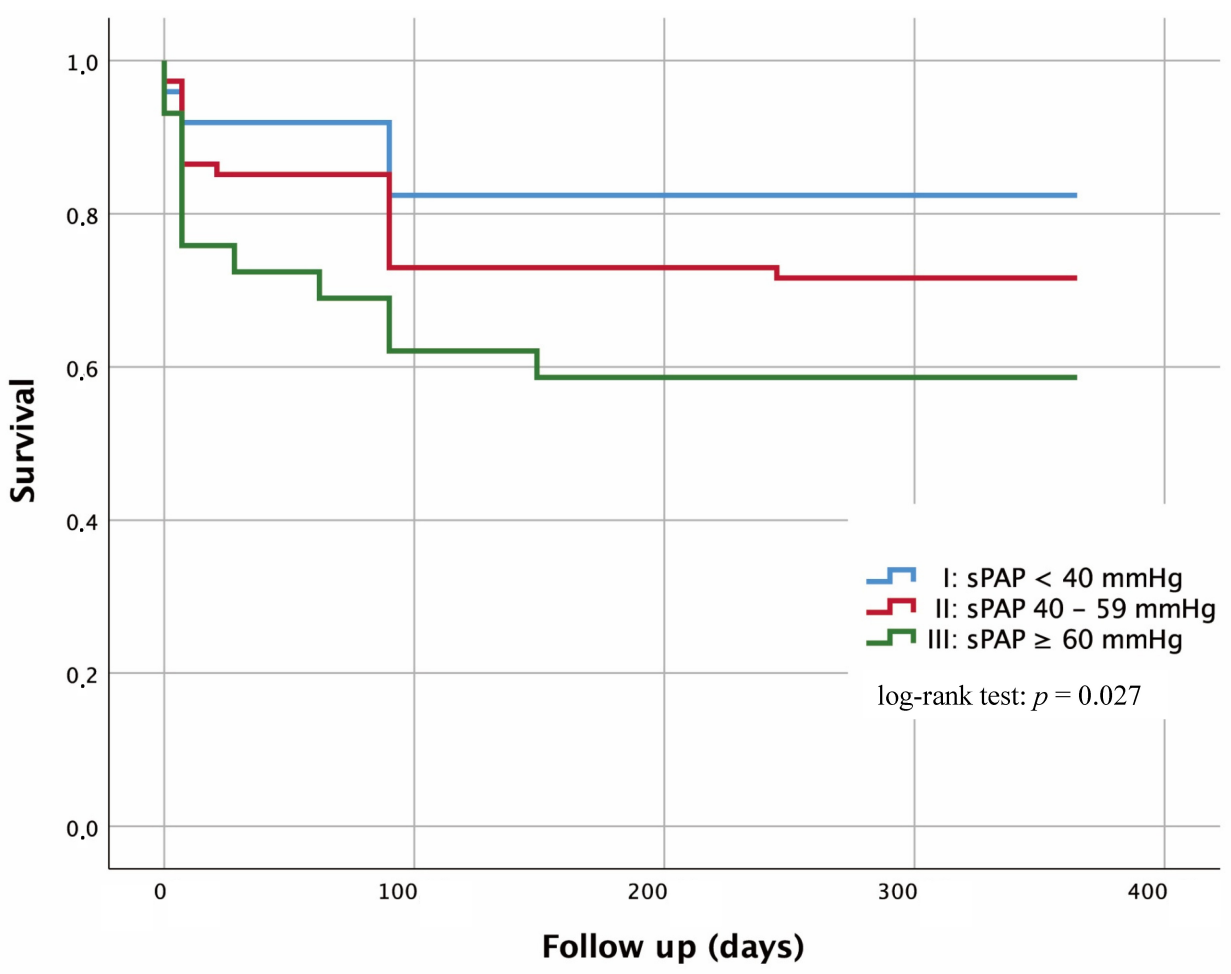
**Kaplan-Meier curves for detection of 1-year survival in dependence of several risk groups**. I: sPAP <40 mmHg; II: sPAP 40–59 mmHg; III: sPAP ≥60 mmHg; Log-rank test I vs. II: *p* = 0.123; I vs. III: *p* = 0.007; II vs. III: *p* = 0.154.

Kaplan-Maier curves were performed with regard to 1-year survival in dependence of severity of sPAP (Fig. 4).

Patients with an sPAP <40 mmHg were classified in group I and, according to current studies, show a generally low risk for the presence of PH. 17.6% of these patients died within one year. In contrast, 28.4% deaths occurred in patients with an sPAP of 40–59 mmHg and thus an intermediate risk for PH (group II). Regarding group I and group II, the log-rank test showed no significant difference with *p* = 0.123. Patients with an sPAP ≥60 mmHg and a pronounced risk for the presence of PH (group III) showed a mortality rate of 41.4% and therefore a significant difference (*p* = 0.007) to group I. In contrast, no relevant significance (*p* = 0.154) could be detected between group II and III.

### 3.7 Cox Proportional Hazard Regression

To investigate several influencing variables concerning 1-year mortality after TAVR, a univariate and multivariate Cox proportional hazard regression was presented (Table 2).

**Table 2.** **Univariate and multivariate Cox regression analysis detecting predictors of 1-year mortality**.

1-year mortality Cox regression analysis	Univariate		Multivariable	
	Hazard Ratio (95% CI)	*p*-value	Hazard Ratio (95% CI)	*p*-value
Age	1.028 (0.978–1.082)	0.278		
Gender (male)	1.148 (0.710–1.856)	0.574		
Weight	1.040 (0.988–1.095)	0.135		
BMI	1.122 (0.960–1.312)	0.149		
NYHA ≥ III	0.787 (0.411–1.509)	0.471		
STS-Score	1.161 (0.869–1.550)	0.313		
Diabetes mellitus	1.042 (0.595–1.827)	0.886		
Arterial Hypertension	1.163 (0.623–2.172)	0.635		
CVD (all)	1.416 (0.797–2.516)	0.235		
CVD - 1 vessel	1.250 (0.714–2.188)	0.435		
CVD - 2 vessels	0.812 (0.325–2.028)	0.656		
CVD - 3 vessels	0.362 (0.113–1.155)	0.086	0.669 (0.075–5.971)	0.719
Myocardial infarction	0.965 (0.236–3.942)	0.960		
Atrial fibrillation	1.376 (0.844–2.242)	0.200		
Pacemaker	1.573 (0.719–3.443)	0.257		
Malignancy	0.866 (0.473–1.586)	0.641		
Stroke	1.028 (0.374–2.825)	0.957		
pAVK	0.690 (0.217–2.198)	0.531		
COPD	1.506 (0.746–3.041)	0.253		
LVEF	0.997 (0.975–1.019)	0.799		
LVEDD	0.940 (0.619–1.428)	0.772		
IVSd	1.158 (1.068–1.257)	<0.001	0.962 (0.662–1.397)	0.838
AV Vmax	0.971 (0.833–1.132)	0.706		
AV dpmean	1.008 (0.989–1.028)	0.403		
AV dpmax	1.002 (0.990–1.015)	0.747		
TAPSE	0.947 (0.832–1.078)	0.412		
sPAP	1.020 (1.004–1.036)	0.015	1.024 (0.985–1.065)	0.238
AVI ≥ II°	0.648 (0.307–1.366)	0.254		
MVI ≥ II°	0.563 (0.288–1.103)	0.094	0.331 (0.056–1.962)	0.223
TVI ≥ II°	0.588 (0.268–1.289)	0.185		
Creatinine	1.403 (0.738–2.666)	0.301		
BNP	0.996 (0.780–1.272)	0.975		
cTnI	1.453 (1.099–1.921)	0.009	1.598 (1.174–2.174)	0.003
Hkt	0.981 (0.935–1.030)	0.445		
Hb	0.967 (0.843–1.108)	0.626		
CK	0.970 (0.746–1.263)	0.823		
sST2	1.178 (0.935–1.483)	0.164		
GDF-15	1.082 (0.854–1.372)	0.513		
H-FABP	1.008 (0.795–1.277)	0.948		
IGF-BP2	1.473 (1.107–1.960)	0.008	1.550 (1.122–2.140)	0.008
suPAR	0.905 (0.694–1.180)	0.461		

BMI, body mass index; CVD, cardiovascular disease; LVEF, left ventricular ejection fraction; LVEDD, left ventricular end-diastolic diameter; IVSd, interventricular septal thickness at diastole; AV Vmax, maximal velocity over aortic valve; AV dpmean, mean pressure gradient over aortic valve; AV dpmax, maximal pressure gradient over aortic valve; TAPSE, tricuspid annular plane systolic excursion; sPAP, systolic pulmonary artery pressure; AVI, aortic valve insufficiency; MVI, mitral valve insufficiency; TVI, tricuspid valve insufficiency; BNP, brain natriuretic peptide; cTnI, cardiac Troponin I; CK, creatine kinase; sST2, soluble suppression of tumorigenicity-2; GDF-15, growth/fifferentiation of factor-15; H-FABP, heart-type fatty-acid binding protein; IGF-BP2, insulin like growth factor binding protein 2; suPAR, soluble urokinase-type plasminogen activator receptor.

The result of univariate analyses showed agreement (*p *< 0.100) with echocardiographic data (diameter of interventricular septum, sPAP and mitral insufficiency ≥II°), with concomitant disease (cardiovascular disease with 3 vessels) and with laboratory data (cTnI and IGF-BP2). After inclusion of these data in a multivariate analysis, cTnI (*p* = 0.003) and IGF-BP2 (0.008) remained as independent factors for increased mortality.

## 4. Discussion

This was one of the first studies on patients with severe AS in which an attempt was made to investigate the context between the expression of cardiovascular biomarkers and the severity of sPAP in order to draw conclusions about the presence of PH.

In several studies of severe AS patients with additional right heart catheter measurements, PH was detected in 48–75%. Echocardiographically, an sPAP ≥40 mmHg is considered to a high probability of PH. In the present study, 97 of 179 patients showed an sPAP ≥40 mmHg which corresponded to a percentage of approximately 54.2% and thus—by the criterion of sPAP alone—is a quite realistic PH proportion in the present collective. Also, the present study represents well that patients with an sPAP ≥40 mmHg are significantly more likely to have severe mitral regurgitation and thus significantly more likely to have atrial fibrillation [26, 27]. Mitral valve insufficiency next to severe AS is a constellation of “double valve disease”, which exerts additional hemodynamic stress on the heart and pulmonary circulation. This may influence the release of cardiovascular biomarkers. In addition, increased atrial fibrillation plays a relevant role in diastolic dysfunction and increased rigidity of the left ventricle. In the current literature [28], a relevant relationship between diastolic dysfunction and echocardiographic sPAP is described, which potentially leads to an additional increase of cardiac biomarkers.

Plasma concentrations of cTnI, BNP, sST2, GDF-15, IGF-BP2, suPAR, and BNP were significantly increased in patients with an sPAP ≥40 mmHg. In a prospective study of 60 patients with severe AS and also echocardiogaphically determined PH, Gumauskienė *et al*. [29] examined NT-proBNP and GDF-15. This working group chose the cut-off for the presence of PH at an sPAP ≥45 mmHg, slightly higher than in the present cohort. Despite different sPAP cut-off values, significantly higher plasma concentrations were detected for both NT-proBNP and GDF-15 in the “PH group” in comparison to the “non-PH group”, consistent with the results of our study. In a publication of 252 patients with severe AS and invasively measured right heart catheterization data to determine PH, Maeder *et al*. [30] described that BNP or its biologically inactive signal peptide NT-proBNP is indicative for the presence of PH at higher plasma concentrations. Again, it must be emphasized that a large proportion of PH patients have atrial fibrillation (46.4%) and mitral valve insufficiency ≥II° (33.0%) as a potential “confounding factor” in the current cohort. This results in a higher degree of diastolic dysfunction and thus would be a possible reason for a consecutive increase in both sPAP and certain cardiovascular biomarkers.

sST2, IGF-BP2, and suPAR were studied for the first time in the constellation with severe AS and echocardiographically detected PH. Geenen *et al*. [31] reported on the expression of sST2 based on patients with different etiologies of PH. They came up with significantly higher levels of sST2 in their collective primarily compared with healthy subjects, but also with higher plasma concentrations depending on the severity of the respective disease process. The excessive release of growth factors (IGF-BP2 in this case) during lung remodeling processes in the setting of post-capillary PH is also not surprising. Yang *et al*. [32] found markedly increased plasma concentrations of IGF-BP2 in two independent pulmonary artery hypertension (PAH) collectives. In conclusion, the levels of suPAR measured here could also be consistent with current literature. Mirna *et al*. [33] provided evidence of increased plasma concentrations of suPAR in particular as a relevant indicator of post-capillary PH.

H-FABP—in contrast to all other biomarkers—did not show significantly increased plasma concentrations in the “PH group”. A possible explanation could be that H-FABP is organotropic and secreted almost exclusively by cardiomyocytes, whereas the other investigated biomarkers are partly produced in several organs by different cell types. Since H-FABP is mainly released during myocardial injuries such as myocardial infarction or acute heart failure, higher plasma levels of H-FABP are also observed in severe AS [34]. However, no additional significant stimulus for cardiomyocytes to secrete greater amounts of H-FABP is created by pulmonary remodeling processes that occur. Nevertheless, a tendency to higher concentrations due to the additional right ventricular load in the context of PH can be detected.

With regard to the results of AUROC analyses, a comparison with Gumauskienė *et al*. [29] is again useful. Their GDF-15 and NT-proBNP analyses showed similar ROC curves as in our study. Because of the higher sPAP value of 45 mmHg, the cut-off value of GDF-15 with 3393 pg/mL was higher than in our group (1117 pg/mL). The BNP cut-off value for the presence of PH was 2248 pg/mL, which is relevantly higher than the cut-off levels (58–190 pg/mL) compiled by Parikh *et al*. [35] for the prediction of symptoms in the setting of severe AS. Here, it is clear how a consequent right heart strain arising from PH influences the secretion of BNP. Regarding sST2, a relevant cut-off value of 16,045 pg/mL was shown. This value was also higher compared to a study of our own working group [36] on patients of another collective with severe AS (cut-off value 10,070 pg/mL for the prediction of 1-year mortality). For H-FABP, no significant cut-off value could be derived based on the hypothesis mentioned above. As significant as almost all biomarker cut-off values in the AUROC analyses may be at first glance, it becomes clear at second glance when considering the respective sensitivities and specificities that their use in clinical routine is not practicable. The use of a solitary biomarker determination with a sensitivity of 51% and a specificity of 76% (as an example sST2 in present study) would not be clinically useful and would only waste resources. Of all biomarkers studied, BNP and cTnI showed the best results with a Youden index of 0.36 each. However, BNP and cTnI are increased in numerous cardiac diseases such as acute or chronic heart failure or cardiomyopathies of any kind [37]. Even severe AS alone without left ventricular decompensation is already a stimulus for increased BNP or cTnI release [30]. Therefore, it is not surprising that the sensitivity and thus the discriminatory power between severe AS without PH and severe AS with PH is not given.

Correlations between sPAP and various cardiovascular biomarkers were performed. The results were sobering. Only for BNP (r: 0.367; *p *< 0.001) and IGF-BP2 (r: 0.324; *p* = 0.003) a moderate correlation and for GDF-15 (r: 0.204; *p* = 0.014) a weak correlation was found, whereas for other biomarkers a correlation was almost non-existent. Regarding the correlation of GDF-15 and sPAP, the working group around Fabiani *et al*. [38] also showed only slightly higher values with an r: 0.36; *p* = 0.001. However, in contrast to our study, the same working group could detect a positive correlation between sST2 and sPAP (r: 0.36; *p* = 0.04) in another publication [39]. Regarding suPAR and IGF-BP2, there have been no relevant comparative studies. A potential hypothesis of low or even absent correlations could be possibly due to the fact that in the present collective, patients with an sPAP ≥40 mmHg were significantly more likely to have moderate to severe tricuspid regurgitation compared to patients with an sPAP <40 mmHg (28.9% vs. 6.1%; *p *< 0.001). Fei *et al*. [40] compared sPAP measured by echocardiography with sPAP measured by right heart catheterization in a study. Here, the largest discrepancies were seen in patients with severe tricuspid regurgitation and severe PH. Fisher *et al*. [41] described in their patient population with the same questioning a general underestimation of sPAP when echocardiographic techniques were used. General underestimation of sPAP in severe tricuspid regurgitation may be due to the fact that the large pendulum volume no longer generates adequate flow acceleration and thus little to no TRV can be derived echocardiographically. This leads consecutively to an underestimation of the sPAP. Therefore, it is even more essential not to rely on the sPAP as the sole criterion for the presence of PH, but to include additional laboratory markers to support the clinical diagnosis.

## 5. Limitation

The present study is based on data from a small cohort over a circumscribed time period (2016–2018). Biomarker levels were only measured at baseline without statement regarding expression after TAVR procedure. Additionally, technical pitfalls in echocardiographic measurements which lead to misclassifications should always be conceded, even if examinations were performed by experienced clinical investigators.

## 6. Conclusions

There is still scarce information about predictors of post-capillary PH in patients with severe AS concerning non-invasive ways. The sPAP is ultimately a solid marker from the echocardiographic side to roughly assess the presence or absence of PH. Nevertheless, correct derivation of TRV is prone to error because it depends on the sound quality and the experience of the examiner. From this point of view, laboratory determinations of cardiovascular biomarkers to concretize possible PH, which is crucial for the long-term survival of patients with severe AS, may possibly provide guidance. Larger study populations with combined biomarker scores are needed to further refine cut-off values. But also the respective expression of singular biomarkers should be investigated with regard to possible “confounders” such as reduction of LVEF, diastolic dysfunction, severity of aortic valve stenosis in dependencies of AV Vmax, AV dpmean, AVdpmax as well as valve opening area.
